# Supplementary material for: Impact of High-Pressure Homogenization on Enhancing the Extractability of Phytochemicals from Agri-Food Residues
Source: Molecules. 2023 Jul 26;28(15):5657. doi: 10.3390/molecules28155657 (PMC10420202; doi:10.3390/molecules28155657)
Supplement: Supplementary file 1 [file molecules-28-05657-s001.zip › molecules-2492050-supplementary.pdf]

molecules

MDPI

Article

Impact of High-Pressure Homogenization on Enhancing the Extractability of Phytochemicals from Agri-Food Residues

Annachiara Pirozzi [1] and Francesco Donsì [1,2,*]

[1] Department of Industrial Engineering, University of Salerno, Via Giovanni Paolo II, 132, 84084 Fisciano, Italy; apirozzi@unisa.it
[2] ProdAl Scarl, University of Salerno, Via Giovanni Paolo II, 132, 84084 Fisciano, Italy
* Correspondence: fdonsi@unisa.it; Tel: +39-089-964135

S1. RSM analysis and optimization of the conventional extraction process

To optimize the recovery of total phenols from agri-food residues using conventional SLE, RSM was used to explore the effects of different organic solvents and their concentrations (Table 3). Figure S1 shows the results of the actual (experimental) values of total phenols against the predicted values of the response surface method.

Citation: Pirozzi, A.; Donsì, F. Impact of High-Pressure Homogenization on Enhancing the Extractability of Phytochemicals from Agri-Food Residues. *Molecules* **2023**, *28*, 5657. https://doi.org/10.3390/molecules28155657

Academic Editors: Federica Ianni and Roccaldo Sardella

Received: 22 June 2023
Revised: 20 July 2023
Accepted: 24 July 2023
Published: 26 July 2023

Copyright: © 2023 by the authors. Licensee MDPI, Basel, Switzerland. This article is an open access article distributed under the terms and conditions of the Creative Commons Attribution (CC BY) license (https://creativecommons.org/licenses/by/4.0/).

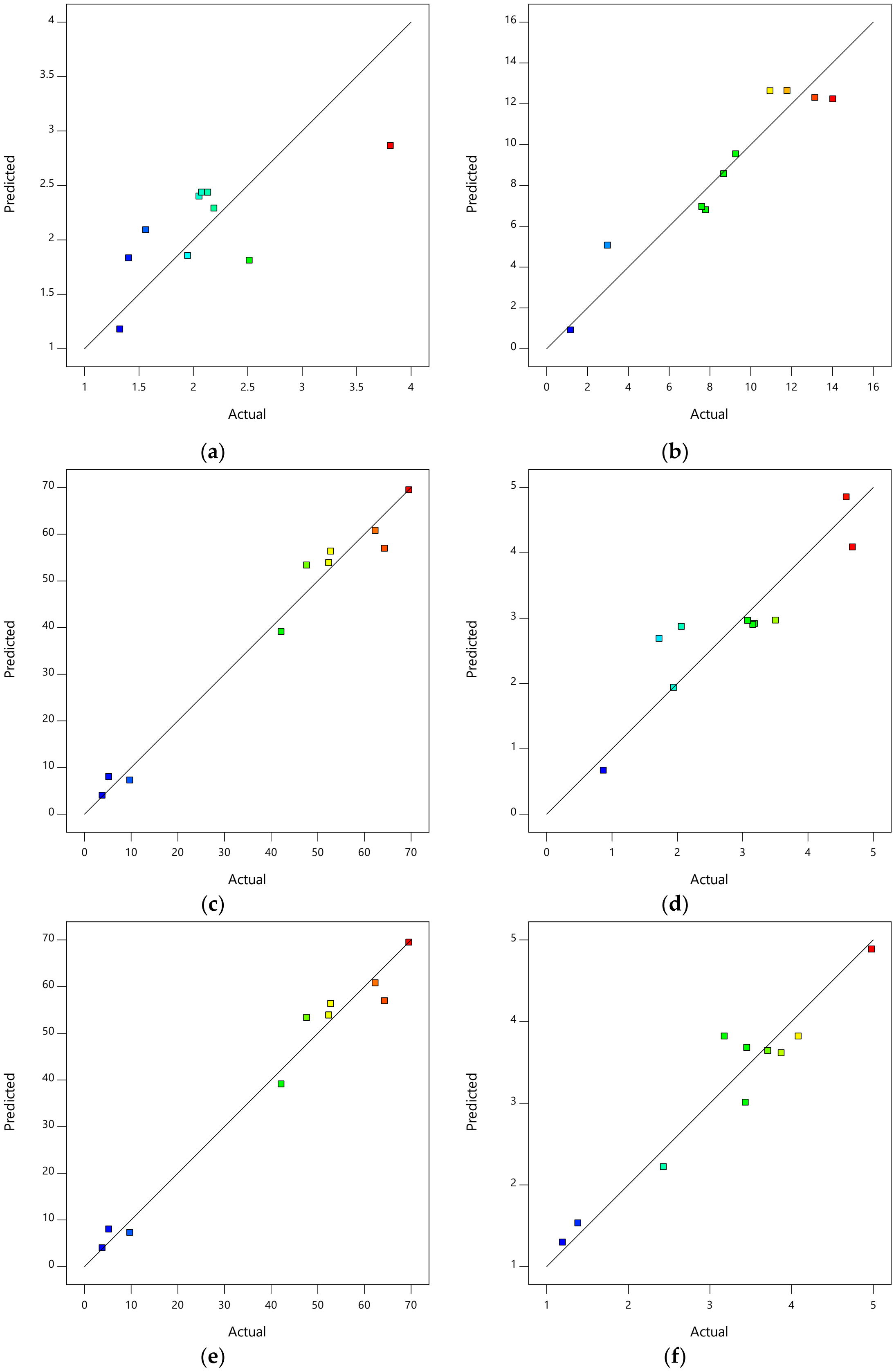

Predicted
Actual
(a)
Predicted
Actual
(b)
Predicted
Actual
(c)
Predicted
Actual
(d)
Predicted
Actual
(e)
Predicted
Actual
(f)

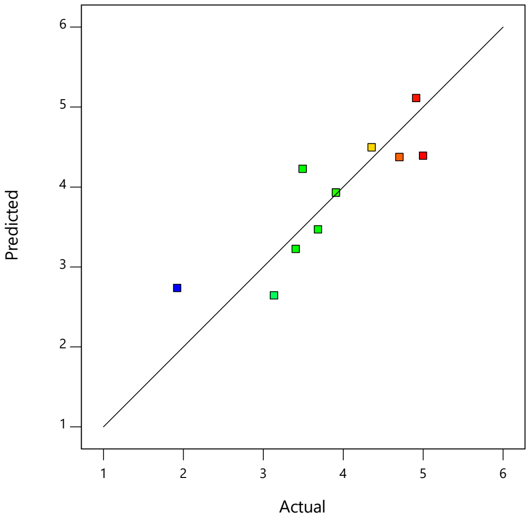

(g)

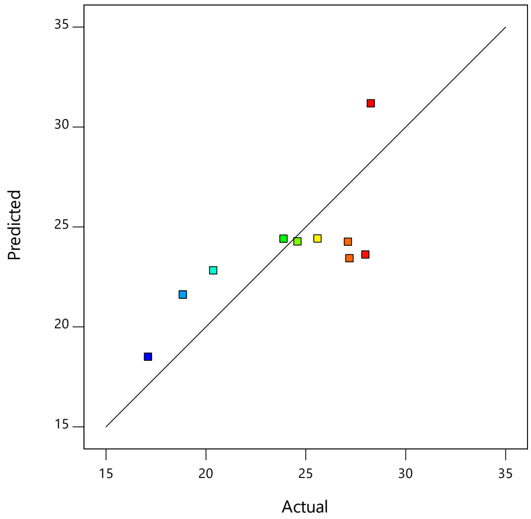

(h)

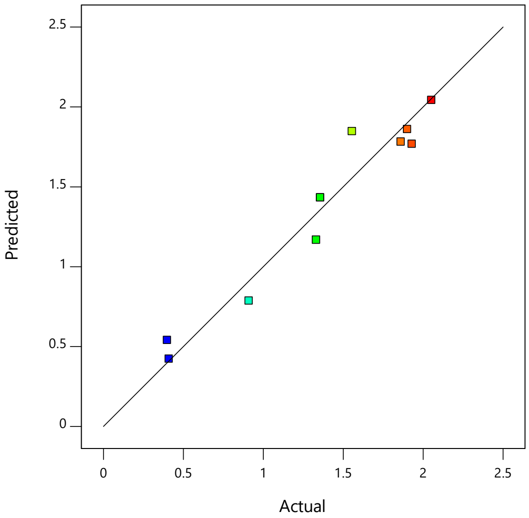

(i)

Figure S1. Scatter plot of predicted value vs actual value from RSM design for different agri-food residues: (**a**) hemp cake; (**b**) sunflower cake; (**c**) roasted coffee beans; (**d**) white grape pomace; (**e**) red grape pomace; (**f**) wheat middlings; (**g**) wheat bran; (**h**) tomato pomace; (**i**) rice husk.

The data obtained from the I-Optimal experiment design were fitted to a second-order polynomial equation (Equation 2) [56].

Table S1. Fitting regression coefficients of second-order polynomial model for the total phenols extraction with SLE technique of different agri-food residues for each organic solvent used.

	**Acetone**			**Ethanol**			**Methanol**		
	β_0	β_1	β_2	β_0	β_1	β_2	β_0	β_1	β_2
Hemp cake	-0,1123	0,0812	-0,0006	-0,1237	0,0766	-0,0006	0,6237	0,0719	-0,0006
Sunflower cake	2,5330	0,3433	-0,0036	2,4329	0,3856	-0,0036	-0,0259	0,4279	-0,0036
Roasted coffee beans	42,3743	1,4393	-0,0182	28,8449	1,6148	-0,0182	10,5710	1,7901	-0,0182
White grape pomace	4,5404	0,0220	-0,0004	0,9837	0,0554	-0,0004	-0,9552	0,0888	-0,0004
Red grape pomace	42,3743	1,4393	-0,0182	28,8449	1,6148	-0,0182	10,5710	1,7902	-0,0182
Wheat middlings	4,2049	0,0448	-0,0007	2,7387	0,0618	-0,0007	1,7278	0,0789	-0,0007
Wheat bran	3,7358	0,0665	-0,0007	2,7903	0,0711	-0,0007	2,2394	0,0757	-0,0007
Tomato pomace	17,8663	0,2388	-0,0011	18,9785	0,1521	-0,0011	22,5353	0,0653	-0,0011
Rice husk	1,1014	0,0470	-0,0005	0,5785	0,0534	-0,0005	0,1858	0,0600	-0,0005

Table S2. Analysis of variance (ANOVA) of the second-order polynomial model for the total phenols extraction with SLE technique of different agri-food residues.

	R^2	Adj-R^2	C.V. (%)
Hemp cake	0.5536	0.4880	33.04
Sunflower cake	0.9194	0.7315	19.29
Roasted coffee beans	0.9780	0.9266	12.10
White grape pomace	0.8285	0.7283	25.19
Red grape pomace	0.9780	0.9266	12.10
Wheat middlings	0.9275	0.7585	13.44
Wheat bran	0.7433	0.5444	16.61
Tomato pomace	0.5575	0.4751	16.36
Rice husk	0.9435	0.8118	14.33

Through analysis of variance, the R^2 values of the models were higher than 0.74 in most of the cases and the Adj-R^2 values did not differ significantly from R^2 values. This indicates that the regression model fitted well with the experimental data, except for hemp cake and tomato pomace (R^2 ~ 0.56). Another parameter that demonstrates the adequacy of the model is the coefficient of variation (C.V., %) which suggests a lower spread of data values relative to their mean.

Furthermore, Figure S2 graphically represent the dependence of total phenols release from solvent concentration, for the three different organic solvent used during the SLE. The ultimate aim of the plots is to enable the prediction of the optimum values of the independent variables (solvent type and solvent concentration) that maximize the response variable (total phenols).

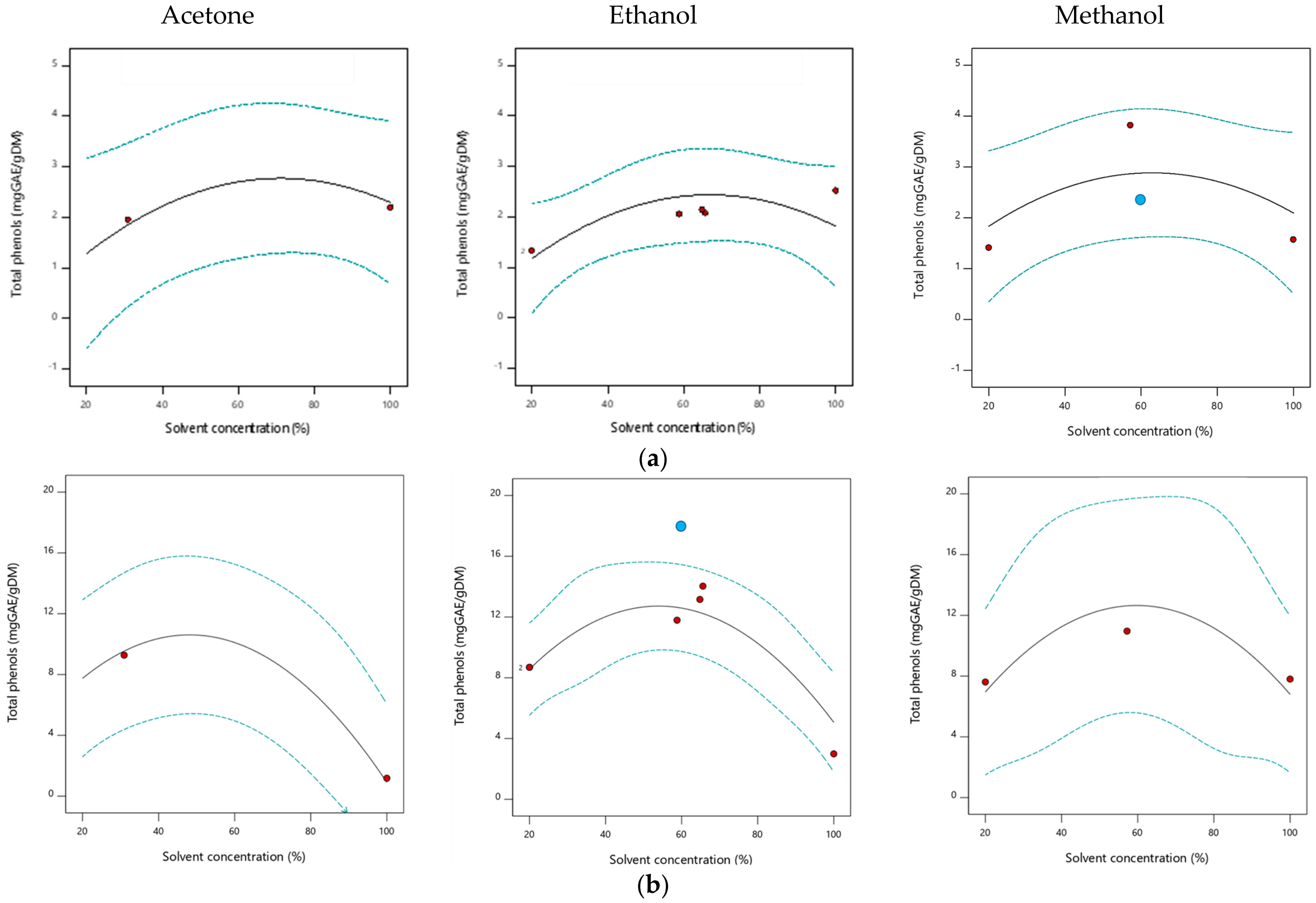

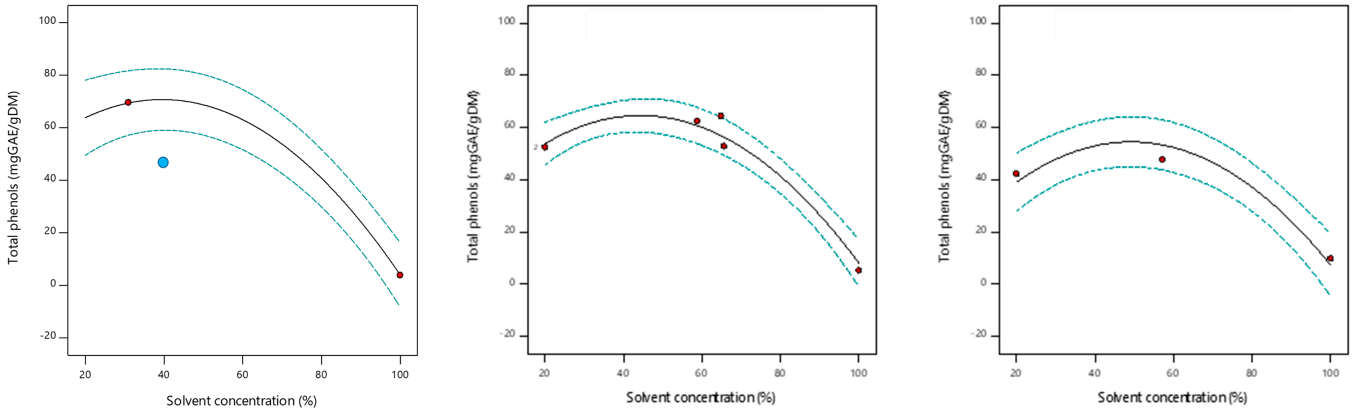
Total phenols (mgGAE/gDM)
Solvent concentration (%)
Total phenols (mgGAE/gDM)
Solvent concentration (%)
Total phenols (mgGAE/gDM)
Solvent concentration (%)

(**c**)

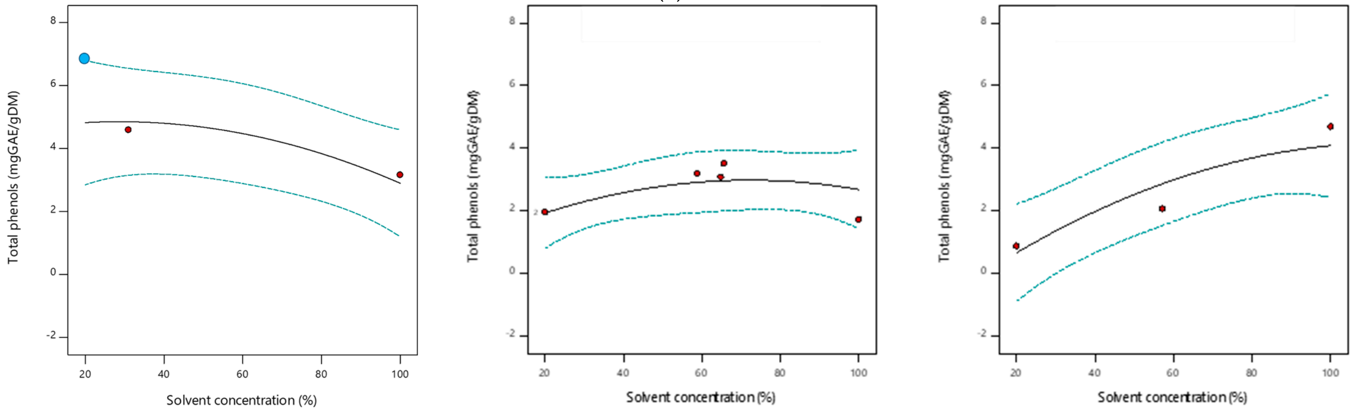
Total phenols (mgGAE/gDM)
Solvent concentration (%)
Total phenols (mgGAE/gDM)
Solvent concentration (%)
Total phenols (mgGAE/gDM)
Solvent concentration (%)

(**d**)

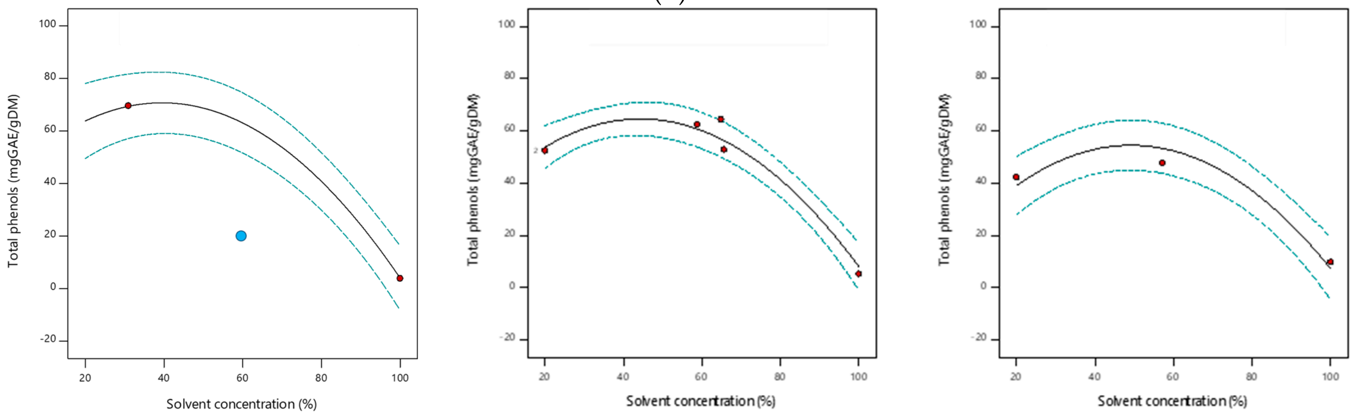
Total phenols (mgGAE/gDM)
Solvent concentration (%)
Total phenols (mgGAE/gDM)
Solvent concentration (%)
Total phenols (mgGAE/gDM)
Solvent concentration (%)

(**e**)

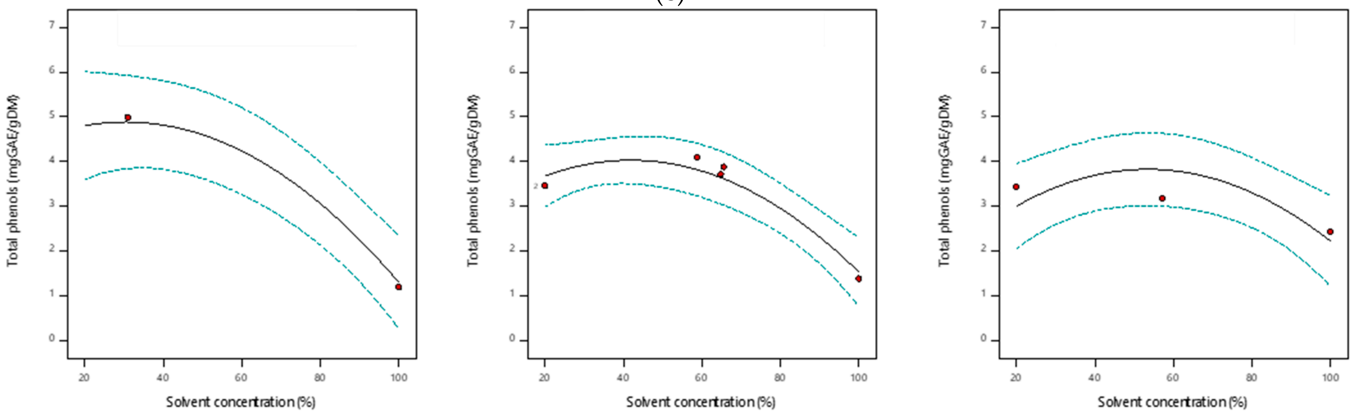
Total phenols (mgGAE/gDM)
Solvent concentration (%)
Total phenols (mgGAE/gDM)
Solvent concentration (%)
Total phenols (mgGAE/gDM)
Solvent concentration (%)

(**f**)

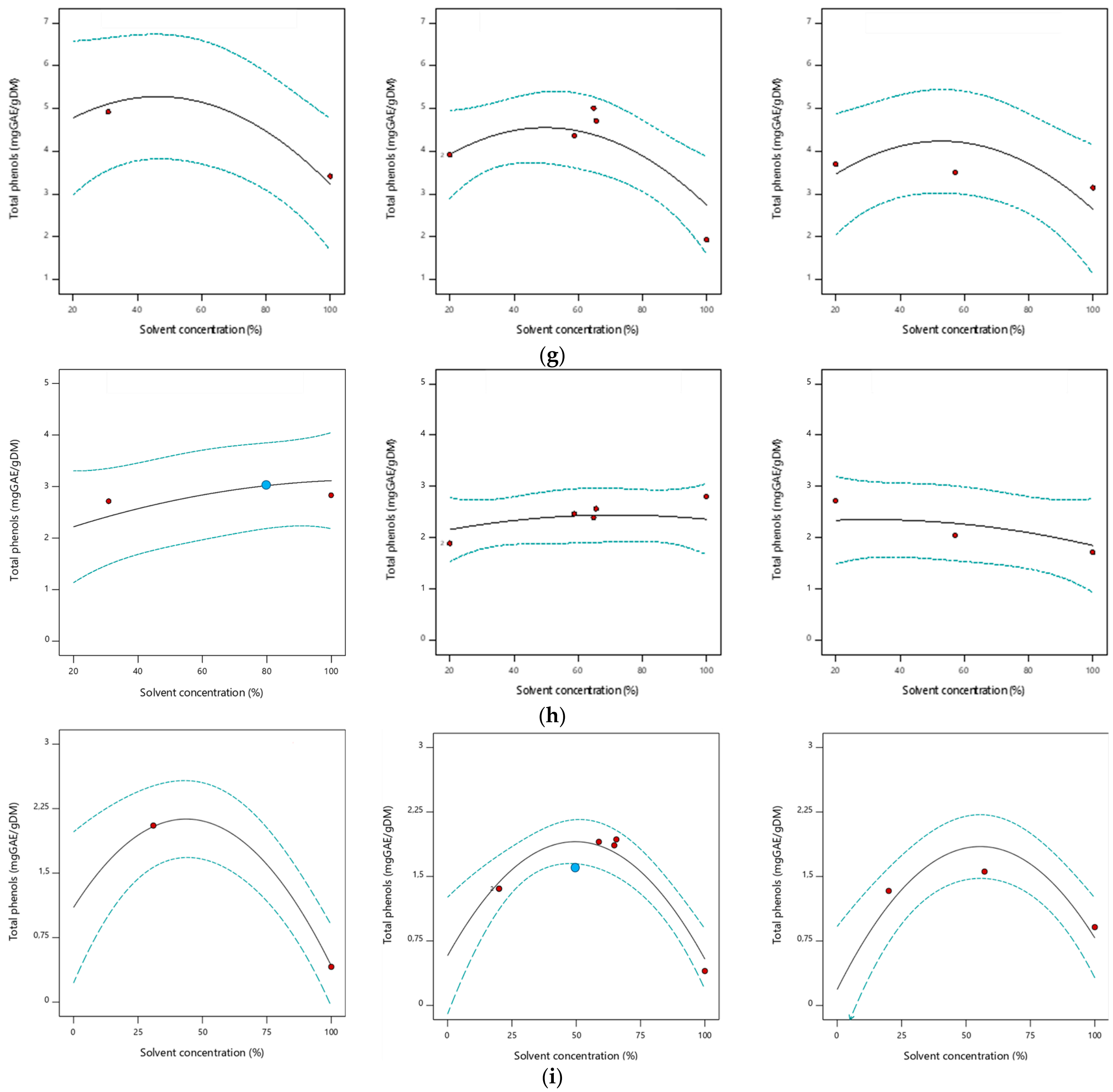

Figure S2. Dependance of the total phenols (mgGAE/gDM) extraction from solvent concentration (%) for each solvent and different agri-food residues: (**a**) hemp cake; (**b**) sunflower cake; (**c**) roasted coffee beans; (**d**) white grape pomace; (**e**) red grape pomace; (**f**) wheat middlings; (**g**) wheat bran; (**h**) tomato pomace; (**i**) rice husk. Dashed blue lines represent the 95% confidence interval bands, red points represent the experimental design points used in model training, and blue points represent independent experimental design points obtained at the optimized operating conditions, hence used for model validation (Table S3).

It was found that pure solvents had a negative effect on the concentration of phenolic compounds in the extracts. More specifically, water-alcohol mixtures were more efficient in extracting phenolic constituents compared to pure solvents, likely due to improved mass transfer facilitated by water's presence. Previous studies have argued that water might cause the swelling of the plant material, facilitating the entry of the solvent into the cell and enhancing extraction yields [57], especially for polar compounds stored in the vacuoles. Therefore, solvent-water mixtures have the capacity to extract more efficiently

the compounds contained in the internal organelles. The polarity of the solvent plays a fundamental role in phenols solubilization, causing higher phenolic concentrations in the extracts [41]. Based on these findings, the optimal solvent and solvent-water mixture ratios were selected (Table S3).

Table S3. Optimal solvent and solvent concentration for the total phenols extraction with SLE technique of different agri-food residues.

	Type of solvent	Solvent-water mixture ratios (% v/v)
Hemp cake	Methanol	60
Sunflower cake	Ethanol	60
Roasted coffee beans	Acetone	40
White grape pomace	Acetone	20
Red grape pomace	Acetone	60
Wheat middlings	Water	-
Wheat bran	Water	-
Tomato pomace	Acetone	80
Rice husk	Ethanol	50

To optimize the extraction time and minimize energy costs, bioactive compound extractability from agri-food residues was investigated over a 24-hour period under the defined optimal extraction conditions. The extraction time is an important parameter to be optimized in order to minimize energy costs of the process. To this purpose, Figure S3 illustrates the kinetics of polyphenols extraction from agri-food residues using the optimal solvent and solvent-water mixture ratios (Table S3) and for a temperature of 25 °C and a solid-solvent ratio of 1:10.

Additionally, the kinetic experimental results obtained under the suggested optimal conditions were utilized to verify and validate the predicted optimum results of the model. To this purpose, the total phenols extracted after 24 hours were recorded and are presented in Figure S2 (blue points in the graphs). It is noteworthy that, for all agri-food residues, the difference in maximum total phenols falls within an acceptable range of 5%, thereby confirming the predicted model is sufficiently accurate within the 95% prediction interval. However, it is important to mention that this acceptable range does not hold true for sunflower cake, roasted coffee beans, and red grape pomace, which exhibited differences of 12%, 10%, and 60%, respectively, when compared to the predicted intervals. These findings indicate the need for further investigation and refinement in the models pertaining to the aforementioned residues.

The kinetics curves were fitted by the Langmuir model (Equation S1).

$$C\left(\frac{mg_{GAE}}{g_{DM}}\right) = C_{Max}\frac{t}{\tau + t} \quad \text{(S1)}$$

where C_{Max} is the saturation concentration of phenols compounds extraction (mg_{GAE}/g_{DM}), t is the extraction time (min), and τ is time at which half of the maximum concentration of total phenols is extracted (min).

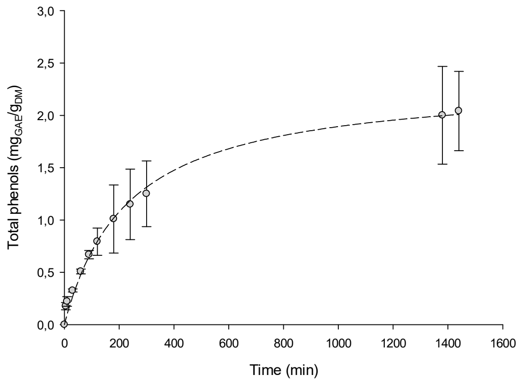
Total phenols (mg_{GAE}/g_{DM})
Time (min)
0,0 0,5 1,0 1,5 2,0 2,5 3,0
0 200 400 600 800 1000 1200 1400 1600

(**a**)

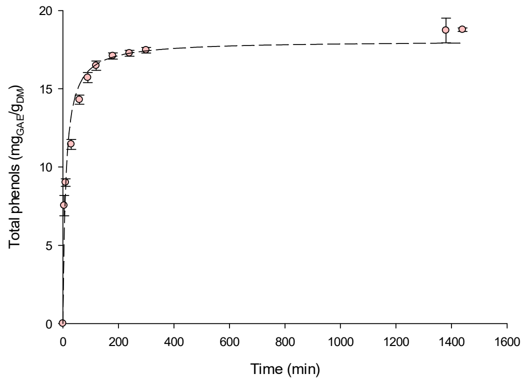
Total phenols (mg_{GAE}/g_{DM})
Time (min)
0 5 10 15 20
0 200 400 600 800 1000 1200 1400 1600

(**b**)

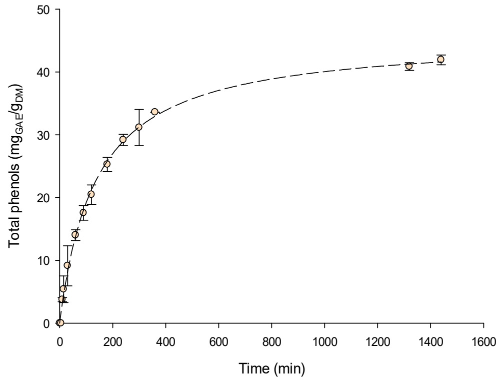
Total phenols (mg_{GAE}/g_{DM})
Time (min)
0 10 20 30 40 50
0 200 400 600 800 1000 1200 1400 1600

(**c**)

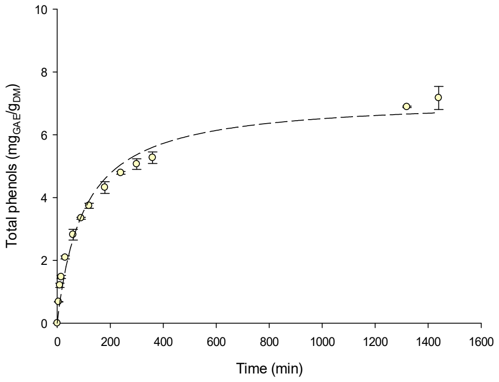
Total phenols (mg_{GAE}/g_{DM})
Time (min)
0 2 4 6 8 10
0 200 400 600 800 1000 1200 1400 1600

(**d**)

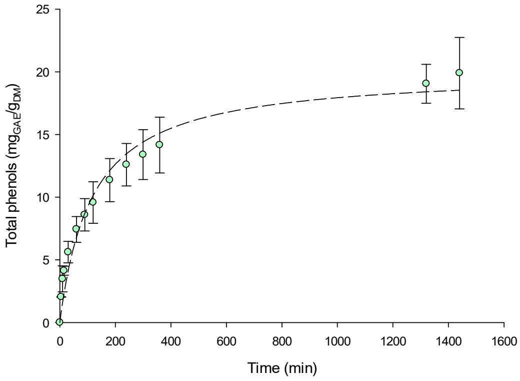
Total phenols (mg_{GAE}/g_{DM})
Time (min)
0 5 10 15 20 25
0 200 400 600 800 1000 1200 1400 1600

(**e**)

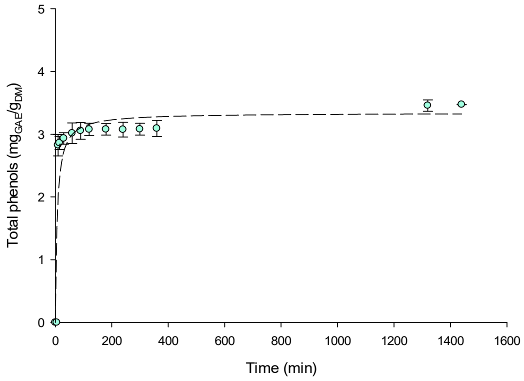
Total phenols (mg_{GAE}/g_{DM})
Time (min)
0 1 2 3 4 5
0 200 400 600 800 1000 1200 1400 1600

(**f**)

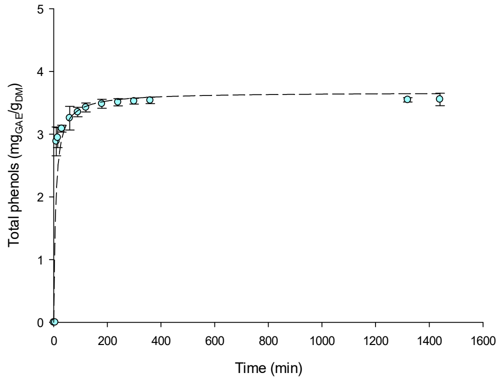
Total phenols (mg_{GAE}/g_{DM})
Time (min)
0 1 2 3 4 5
0 200 400 600 800 1000 1200 1400 1600

(**g**)

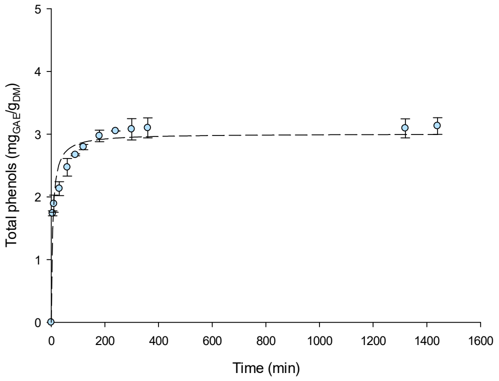
Total phenols (mg_{GAE}/g_{DM})
Time (min)
0 1 2 3 4 5
0 200 400 600 800 1000 1200 1400 1600

(**h**)

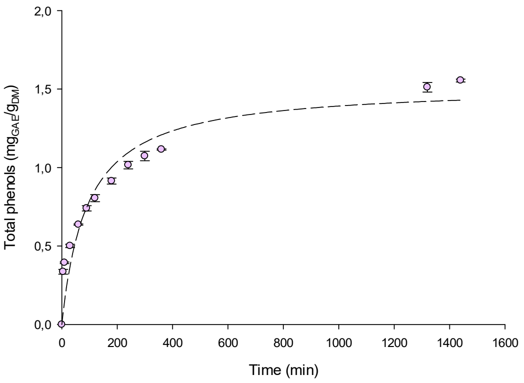

(**i**)

Figure S3. Extraction kinetics of total phenols at 25 °C and solid-solvent ratio 1:10 for different agri-food residues: (**a**) hemp cake with 60 v% methanol; (**b**) sunflower cake with 60 v% ethanol; (**c**) roasted coffee beans with 40 v% acetone; (**d**) white grape pomace with 20 v% acetone; (**e**) red grape pomace with 60 v% acetone; (**f**) wheat middlings with water; (**g**) wheat bran with water; (**h**) tomato pomace with 80 v% acetone; (**i**) rice husk with 50 v% ethanol. Dotted lines represent the Langmuir fitting curve (Equation S1).

Regardless of the sample being investigated, the goodness of fit of the Langmuir kinetic models for all experimental data was confirmed by the values of the coefficients of determination, as shown in Table S4.

Table S4. Fitting parameters and coefficients of determination for the fitting of the data of Figure S3, using Equation S1.

	C_{Max} (mg$_{GAE}$/g$_{DM}$)	τ (min)	R^2	Adj-R^2
Hemp cake	2.333	232.711	0.991	0.909
Sunflower cake	18.057	10.943	0.974	0.971
Roasted coffee beans	45.579	137.989	0.998	0.997
White grape pomace	7.169	100.811	0.975	0.973
Red grape pomace	20.039	117.523	0.964	0.961
Wheat middlings	3.339	6.909	0.807	0.791
Wheat bran	3.666	7.767	0.845	0.832
Tomato pomace	3.007	5.698	0.952	0.947
Rice husk	1.523	93.498	0.917	0.909

It could be observed that for all the residues tested, polyphenols extraction increased continuously with time. The rate of extraction progressively decreased over time reaching an asymptotic value after 24 h of extraction. The extraction curves clearly show that the content of polyphenols greatly varied among the different agri-food residues. Additionally, the differences in particle sizes affected the kinetics of polyphenols extraction. The results obtained by extraction from the agri-food residues with the finest size, wheat middlings (Figure S3.f) and wheat bran (Figure S3.g), which were characterized by mean particle sizes < 0.1 mm and < 0.5 mm, respectively, confirm that complete extraction can be accomplished with shorter extraction times for finer materials. The higher contact surface between the solvent and the fine solid particles favored the extraction of polyphenols. In the case of wheat bran and wheat middlings, the asymptotic polyphenols concentration was reached within the first hour of extraction.

S2. Effect of HPH on extraction efficiency of total phenols

The extraction efficiency of total phenols was investigated by subjecting the agri-food residues to HPH treatment. The results revealed that the application of HPH significantly

enhanced the extraction efficiency of total phenols compared to conventional extraction methods for different agri-food residues, including roasted coffee beans, wheat middlings, wheat bran, and tomato pomace (Figure S4). Quantitative analysis demonstrated a substantial increase (~20%) in the extraction of phenolic compounds following HPH treatment. This finding suggests that the mechanical disruption caused by HPH treatment effectively promotes the release and solubilization of phenolic compounds, thereby enhancing the overall extraction efficiency of total phenols.

These findings contribute to the development of sustainable and efficient extraction methods, which can valorize agri-food residues and increase the availability of bioactive compounds for various applications in the food, pharmaceutical, and cosmetic industries.

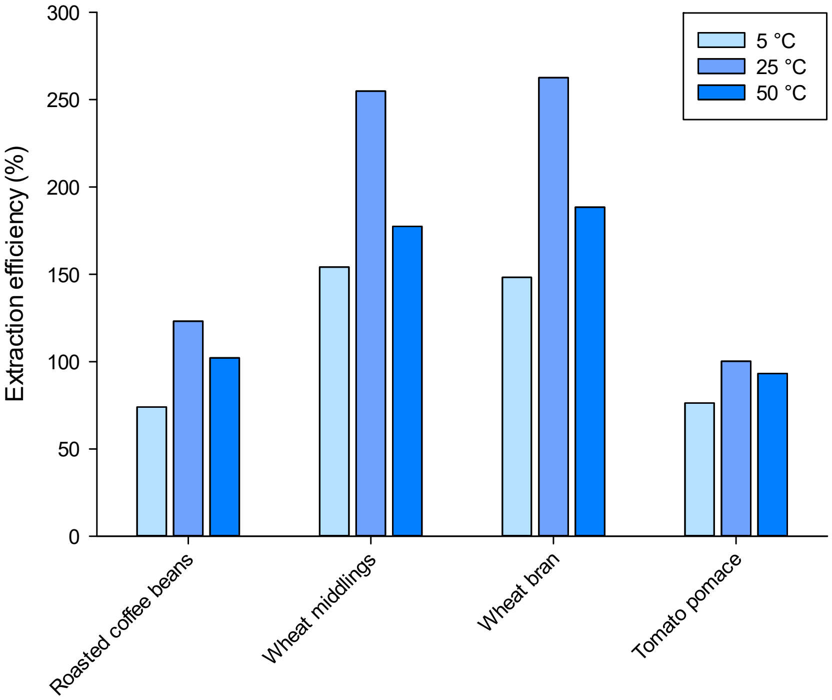

Figure S4. Extraction efficiency of HPH-assisted extraction over conventional SLE for different source based on total phenols.

References

41. Galvan D'Alessandro, L.; Kriaa, K.; Nikov, I.; Dimitrov, K. Ultrasound assisted extraction of polyphenols from black chokeberry. *Sep. Purif. Technol.* **2012**, 93, 42–47, doi:10.1016/j.seppur.2012.03.024.
56. Ratnawati, R.; Wulandari, R.; Kumoro, A.C.; Hadiyanto, H. Response Surface Methodology for Formulating PVA/Starch/ Lignin Biodegradable Plastic. *Emerg. Sci. J.* **2022**, 6, 238–255, doi:10.28991/ESJ-2022-06-02-03.
57. Both, S.; Chemat, F.; Strube, J. Extraction of polyphenols from black tea - Conventional and ultrasound assisted extraction. *Ultrason. Sonochem.* **2014**, 21, 1030–1034, doi:10.1016/j.ultsonch.2013.11.005.

Disclaimer/Publisher's Note: The statements, opinions and data contained in all publications are solely those of the individual author(s) and contributor(s) and not of MDPI and/or the editor(s). MDPI and/or the editor(s) disclaim responsibility for any injury to people or property resulting from any ideas, methods, instructions or products referred to in the content.
